# Clinicopathological Features of Craniopharyngioma: A 15-Year Study From a Tertiary Care Center in Pakistan

**DOI:** 10.7759/cureus.14153

**Published:** 2021-03-28

**Authors:** Saroona Haroon, Anoshia Afzal, Shamail Zia, Syed J Ali, Fazail Zia, Farozaan Shamail, Muhammad Irfan, Atif A Hashmi

**Affiliations:** 1 Pathology, King's Mill Hospital - Sherwood Forest Hospitals, NHS Foundation Trust, Ashfield, GBR; 2 Pathology, Prince Faisal Oncology Centre, King Fahad Specialist Hospital, Buraidah, SAU; 3 Pathology, Aga Khan University, Karachi, PAK; 4 Pathology, University of Oklahoma Health Sciences Center, Oklahoma City, USA; 5 Pathology, Ziauddin University, Karachi, PAK; 6 Pathology, Dow University of Health Sciences, Karachi, PAK; 7 Pathology, Jinnah Sindh Medical University, Karachi, PAK; 8 Statistics, Liaquat National Hospital and Medical College, Karachi, PAK; 9 Pathology, Liaquat National Hospital and Medical College, Karachi, PAK

**Keywords:** craniopharyngioma, papillary craniopharyngioma, adamantinomatous craniopharyngioma

## Abstract

Introduction

Craniopharyngiomas (CPs) are benign neoplasms and most common suprasellar tumors. They are more frequent in children, contributing to a significant number of intracranial tumors in the pediatric population and are thought to be arising either from the epithelial remnant cells of the craniopharyngeal duct or from the adenohypophysis epithelium. Two subtypes of CPs exist, namely, adamantinomatous craniopharyngioma (ACP) and papillary craniopharyngioma (PCP). ACP is more common in children with a relatively aggressive clinical course and more frequent relapses than PCP. The study objective was to evaluate the clinicopathological features of CP in our population.

Methods

We conducted a retrospective observational study in the Department of Histopathology at Aga Khan Hospital, Karachi, Pakistan, over a period of 15 years, from January 2001 to December 2015. All CP cases were included in the study. A total of 207 cases were diagnosed during this period by histopathologists based on histologic features. All slides were retrieved, and diagnosis was confirmed after a reexamination of slides.

Results

We found that the mean age of diagnosis was 25.59±14.71 years, and the median follow-up time was 7 (3-19) years. The number of male patients was 136 (65.7%) and the number of female patients was 71 (34.3%). The most common tumor site was suprasellar (71.5%) followed by the sellar and temporal lobe (12.1% and 6.8%, respectively). The most common complaints were headache (21.7%), followed by loss of vision/decreased vision (16.4%) and vomiting (5.3%). The overall survival rate was 95.2% with a recurrence rate of 5.8%. A significant association of survival was noted with tumor recurrence.

Conclusion

CP is a rare brain tumor with good overall survival. We found a low recurrence rate of CP in our study. However, recurrence was found to be the most important factor determining survival in patients with CP.

## Introduction

Craniopharyngioma (CP) is a tumor of sellar and suprasellar areas with two histologic subtypes. The adamantinomatous craniopharyngioma (ACP) subtype is usually seen in childhood and is more common, whereas the papillary craniopharyngioma (PCP) subtype is almost exclusive to the adult population [1]. The two subtypes differ in their clinical behavior and overall features. The papillary type is usually indolent, whereas the adamantinomatous type has a relatively aggressive clinical course [2]. The most reported symptoms are headache, decreased/loss of vision, and neuroendocrine abnormalities (hormonal changes, personality changes, etc.). The incidence from recent studies appears to be high in males compared to females [3]. However, previous studies had shown an equal incidence in males and females. Malignant transformation is infrequent and usually occurs after multiple recurrences of CP [4]. ACP is relatively common and tends to recur more frequently than PCP whereas PCP has a benign clinical course with less frequent relapses. The usual treatment approach for both subtypes is resection followed by radiotherapy. The study objective was to evaluate the clinicopathological features of CP in our population.

## Materials and methods

**Figure 1:**
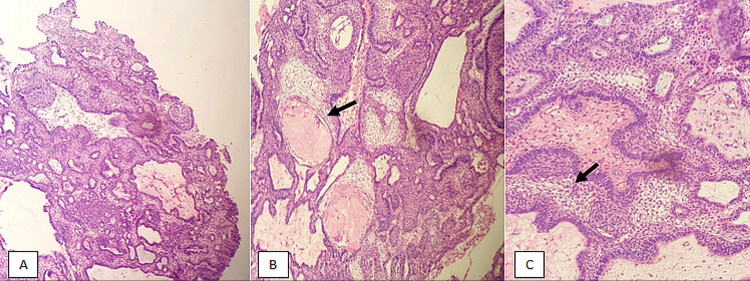
Craniopharangioma, adamantinomatous subtype: (A) H&E-stained section at 40x magnification showing sheets and trabeculae of squamous epithelium; (B) H&E-stained section at 100x magnification revealing wet keratin (arrow); (C) H&E-stained section at 400x magnification depicting stellate reticulum (arrow) H&E, hematoxylin and eosin

We conducted a retrospective observational study in the Department of Histopathology at Aga Khan Hospital (Karachi, Pakistan) over a period of 15 years, from January 2001 to December 2015. All CP cases were included in the study. A total of 207 cases were diagnosed during this period by histopathologists based on histological features. All slides were retrieved, and diagnosis was confirmed after a reexamination of slides. All specimens were received in the histopathology lab. After gross examination and specimen measurement, the tissues were submitted entirely for histopathological examination. A diagnosis of CP was rendered based on histopathological findings. PCP was characterized by papillary (with fibro-vascular cores) and cauliflower-like morphology, composed of non-keratinizing squamous epithelium. Alternatively, ACP was defined by trabeculae and sheets, with nuclear palisading and stellate reticulum. Nodules of anucleated squames (wet keratin) are characteristic of ACP (Figure 1).

Data analysis was performed using Statistical Package for the Social Sciences, Version 26.0 (IBM Corp, Armonk, USA). Independent t-test and Fisher’s exact tests were used to check the association. Survival analysis was done by the Kaplan-Meier method. P-values < 0.05 were considered as significant.

## Results

We reviewed a total of 207 cases and determined their clinicopathological features. The variables that were studied included age, site, symptoms, gender, tumor subtype, recurrence, and survival. We found that the mean age of diagnosis was 25.59±14.71 years, and the median follow-up time was 7 (3-19) years. There were 136 (65.7%) male patients and 71 (34.3%) female patients. The most common site was the suprasellar (71.5%), followed by the sellar and temporal lobe (12.1% and 6.8%, respectively). The most common complaints were headache (21.7%) followed by loss of vision/decreased vision (16.4%) and vomiting (5.3%). The overall survival rate was 95.2% with a recurrence rate of 5.8% (Table 1).

**Table 1:** Clinicopathological features of the population under study SD, standard deviation

Clinicopathologic characteristics	Values
Age (years), mean±SD	25.59±14.71
Follow-up (years), median (range)	7 (3–19)
Gender	
Male, n (%)	136 (65.7)
Female, n (%)	71 (34.3)
Site	
Supraseller, n (%)	148 (71.5)
Sellar, n (%)	25 (12.1)
Frontal, n (%)	11 (5.3)
Sellar and suprasellar, n (%)	9 (4.3)
Temporal, n (%)	14 (6.8)
Headache	
Yes, n (%)	45 (21.7)
No, n (%)	162 (78.3)
Vomiting	
Yes, n (%)	11 (5.3)
No, n (%)	196 (94.7)
Decreased/loss of vision	
Yes, n (%)	34 (16.4)
No, n (%)	173 (83.6)
Fever	
Yes, n (%)	5 (2.4)
No, n (%)	202 (97.6)
Generalized weakness	
Yes, n (%)	5 (2.4)
No, n (%)	202 (97.6)
Tumor type	
Papillary, n (%)	2 (1)
Adamantinomatous, n (%)	205 (99)
Recurrence	
Yes, n (%)	12 (5.8)
No, n (%)	195 (94.2)
Survival status	
Alive, n (%)	197 (95.2)
Expired, n (%)	10 (4.8)

No significant association of clinicopathological features with recurrence was noted (Table 2).

**Table 2:** Association of clinicopathologic characteristics of craniopharangioma with tumor recurrence SD, standard deviation *Independent t-test was applied. **Fisher’s exact test was applied.

Clinicopathologic characteristics	Values		P-value
	Recurrence		
	Yes (n=12)	No (n=195)	
Age (years)*, mean±SD	28.00±19.43	25.44±14.42	0.205
Gender**			
Male, n (%)	8 (66.7)	128 (65.6)	1.000
Female, n (%)	4 (33.3)	67 (34.4)	
Site**			
Supraseller, n (%)	9 (75)	139 (71.3)	0.606
Sellar, n (%)	1 (8.3)	24 (12.3)	
Frontal, n (%)	0 (0)	11 (5.6)	
Sellar and suprasellar, n (%)	0 (0)	9 (4.6)	
Temporal, n (%)	2 (16.7)	12 (6.2)	
Headache**			
Yes, n (%)	3 (25)	42 (21.5)	0.726
No, n (%)	9 (75)	153 (78.5)	
Vomiting**			
Yes, n (%)	0 (0)	11 (5.6)	1.000
No, n (%)	12 (100)	184 (94.4)	
Decreased/loss of vision**			
Yes, n (%)	2 (16.7)	32 (16.4)	1.000
No, n (%)	10 (83.3)	163 (83.6)	
Fever**			
Yes, n (%)	0 (0)	5 (2.6)	1.000
No, n (%)	12 (100)	190 (97.4)	
Generalized weakness**			
Yes, n (%)	1 (8.3)	4 (2.1)	0.260
No, n (%)	11 (91.7)	191 (97.9)	
Tumor type**			
Papillary, n (%)	0 (0)	2 (1)	1.000
Adamantinomatous, n (%)	12 (100)	193 (99)	
Survival status**			
Alive, n (%)	10 (83.3)	187 (95.9)	0.107
Expired, n (%)	2 (16.7)	8 (4.1)	

Similarly, no significant association of survival status was noted with clinicopathological features (Table 3).

**Table 3:** Association of clinicopathologic characteristics of craniopharyngioma with survival status SD, standard deviation *Independent t-test was applied. **Fisher’s exact test was applied.

Clinicopathologic characteristics	Values		P-value
	Survival status		
	Alive (n=197)	Expired (n=10)	
Age (years)*, mean±SD	25.79±14.65	21.60±16.13	0.380
Gender**			
Male, n (%)	128 (65)	8 (80)	0.499
Female, n (%)	69 (35)	2 (20)	
Site**			
Supraseller, n (%)	138 (70.1)	10 (100)	0.673
Sellar, n (%)	25 (12.7)	0 (0)	
Frontal, n (%)	11 (5.6)	0 (0)	
Sellar and suprasellar, n (%)	9 (4.6)	0 (0)	
Temporal, n (%)	14 (7.1)	0 (0)	
Headache**			
Yes, n (%)	43 (21.8)	2 (20)	1.000
No, n (%)	154 (78.2)	8 (80)	
Vomiting**			
Yes, n (%)	10 (5.1)	1 (10)	0.428
No, n (%)	187 (94.9)	9 (90)	
Decreased/loss of vision**			
Yes, n (%)	32 (16.2)	2 (20)	0.670
No, n (%)	165 (83.8)	8 (80)	
Fever**			
Yes, n (%)	5 (2.5)	0 (0)	1.000
No, n (%)	192 (97.5)	10 (100)	
General weakness**			
Yes, n (%)	5 (2.5)	0 (0)	1.000
No, n (%)	192 (97.5)	10 (100)	
Tumor type**			
Papillary, n (%)	2 (1)	0 (0)	1.000
Adamantinomatous, n (%)	195 (99)	10 (100)	
Recurrence**			
Yes, n (%)	9 (4.6)	1 (10)	0.397
No, n (%)	188 (95.4)	9 (90)	

**Figure 2:**
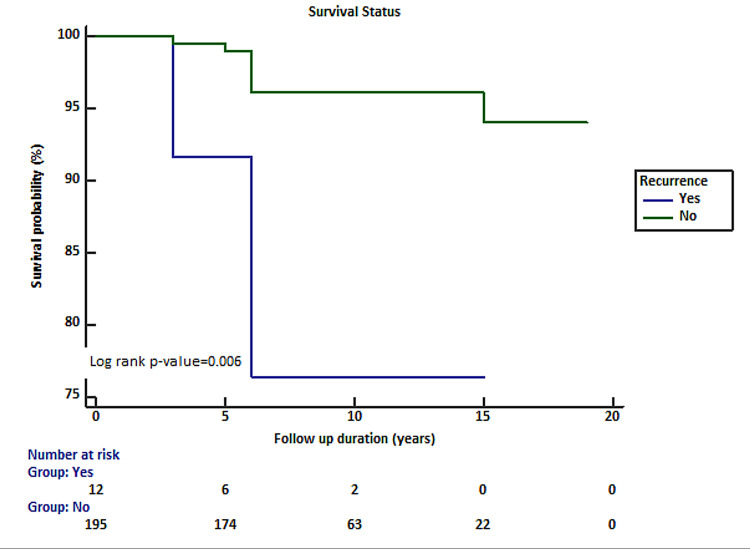
Association of recurrence with survival status in patients with craniopharyngioma

Alternatively, significant association of survival was noted with tumor recurrence (Figure 2).

## Discussion

CP is the most frequent neoplasm of suprasellar and sellar region with ACP being approximately nine times more prevalent than PCP [1,2]. Some studies from Pakistan have found the incidence to be greater in the male population [3], but overall CP affects both males and females equally [1-3]. Malignant transformation occurs in CP, especially after multiple recurrences, and some of the reported cases were treated with radiotherapy [4]. Malignant transformation was reported in patients with giant sellar masses and it is recommended to take extra caution if the size of the lesion is large [4].

The size of PCP is usually smaller and the recurrence rate is lower than ACP [5]. Sanford reported the results of a survey and suggested a very good overall outcome in children who were treated with limited surgery and irradiation compared to the attempted total resection [6]. Wara et al. also reported a 77% 10-year disease-free survival after treatment with radiation and surgery, and advised to follow the patients who underwent total resection without radiation [7].

Schoenfeld et al. also reported better overall outcomes with surgery and radiation compared to gross total resection (GTR) [8]. Other studies also reported that the incidences of endocrinopathies were significantly more with GTR compared with subtotal resection (STR) and radiation [9]. Recent studies have shown that the history of radiation therapy (instead of surgery) is more effective in reducing the resection extent in recurrent cases of craniopharyngioma [10,11].

Based on location and size, differential diagnosis of CP includes pituitary adenoma, epidermoid cyst, and Rathke cleft cyst with squamous metaplasia. Pituitary adenoma is characterized by sheets of monotonous round cells with neuroendocrine appearance. An epidermoid cyst is unilocular lined by keratinizing squamous epithelium. Finally, a Rathke cleft cyst also contains ciliated or mucinous lining that helps in differentiation. In addition, ACPs sometimes show reactive gliosis (piloid gliosis), which sometimes causes confusion and may lead to an erroneous diagnosis of pilocytic astrocytoma. However, the presence of wet keratin and squamous epithelium helps in the correct diagnosis. There is limited role of immunohistochemistry (IHC) in the diagnosis of CP, although ACP is positive with CK7, CK8, CK19 and beta-catenin. PCP is positive with BRAF V600E IHC, while ACP is negative. As far as molecular genetics is concerned, ACP is characterized by mutations that activate Wnt pathway gene CTNNB1 encoding β-catenin in most of the cases, while PCP is characterized by BRAF V600E mutation in more than 90% of cases [1,2,6].

CPs tend to recur and can cause significant mortality and morbidity due to their critical location and hormonal imbalances, and therefore the recommendation is to prefer STR and radiation therapy over GTR [12].

The limitations of our study included a retrospective study design and limited sample size, especially cases of PCP, and therefore, the association of tumor type with recurrence and survival could not be determined. In addition, lack of availability of radiological findings is yet another limitation of the study. Moreover, data regarding GTR versus STR were not available to evaluate the outcome in different treatment groups.

## Conclusions

CP is a rare suprasellar brain tumor with distinguished histological features. In our study, the ACP subtype of CP was far more frequent than the PCP subtype. We found an overall low recurrence rate and good survival in patients of CP in our study. We also noted a significant association of disease recurrence of CP with survival in our study. However, more studies are required to have a better idea of clinical course and prognosis of CP in Pakistan, especially the outcome in different treatment groups, for instance, GTR versus STR with radiation.
